# Maternal Mood and Perception of Infant Temperament at Three Months Predict Depressive Symptoms Scores in Mothers of Preterm Infants at Six Months

**DOI:** 10.3389/fpsyg.2022.812893

**Published:** 2022-01-26

**Authors:** Grazyna Kmita, Eliza Kiepura, Alicja Niedźwiecka

**Affiliations:** ^1^Faculty of Psychology, University of Warsaw, Warsaw, Poland; ^2^Institute of Mother and Child, Warsaw, Poland

**Keywords:** postpartum depression, preterm infants, mothers, fathers, temperament

## Abstract

Postpartum depression is more prevalent in mothers and fathers of preterm infants compared to parents of full-term infants and may have long-term detrimental consequences for parental mental health and child development. The temperamental profile of an infant has been postulated as one of the important factors associated with parental depressiveness in the first months postpartum. This study aimed to examine the longitudinal relationship between depressive symptoms and perceived infant temperament at 3 months corrected age, and depressive symptoms at 6 months corrected age among mothers and fathers of infants born preterm. We assessed 59 families with infants born before the 34th gestational week using the Edinburgh Postnatal Depression Scale (EDPS) and the Infant Behavior Questionnaire-Revised. We found that mothers’ scores on EPDS and infants’ Orienting/regulation at 3 months corrected age predicted mothers’ EPDS scores at 6 months corrected age. In particular, higher depressive scores were related to higher depressive symptoms at 6 months corrected age, whereas higher infant Orienting/regulation was related to lower depressive symptoms at 6 months corrected age. Due to the low internal consistency of EPDS at 6 months for fathers, we were unable to conduct similar analyses for fathers. Our results point to the importance of considering both early indices of maternal mood as well as mother-reported measures of preterm infant temperament in the attempts to predict levels of maternal depressiveness in later months of an infant’s life. Further studies are urgently needed in order to better understand the associations between depressiveness and infant temperament in fathers, and with more consideration for the severity of the effects of infant prematurity.

## Introduction

Despite rapid advances in obstetric and neonatal care, prematurity, i.e., a birth before the 37th gestational week, remains a major health and developmental risk factor for affected children and contributes to increased distress in their parents (Wolke et al., 2019). Multiple emotional reactions of parents to their preterm infants’ hospitalization in a neonatal intensive care unit have been described, including feelings of helplessness and being out of control, uncertainty as to the infant’s survival and health status, sadness, and extreme distress (Lasiuk et al., 2013; Trumello et al., 2018). Studies on the impact of preterm birth on parental mental health point to increased risk of depression and post-traumatic stress disorder as well as increased levels of anxiety and parenting stress, especially in mothers of preterm infants (Kersting et al., 2004; Karatzias et al., 2007; Vigod et al., 2010; Helle et al., 2015; Pace et al., 2016; Anderson and Cacola, 2017; Yildiz et al., 2017; Garfield et al., 2021; Genova et al., 2022). The rates of paternal depression and anxiety seem to be lower than maternal ones, but still elevated when compared to fathers of full-term infants (Treyvaud, 2014; McMahon et al., 2020; Weigl et al., 2020; Baldoni et al., 2021; Genova et al., 2022).

Literature on longer-term trajectories of perinatal depression in parents of preterm infants is still limited and existing results are mixed. Some studies have found no evidence for prolonged risks for parental mental health, at least considering the trajectories of parenting stress (Schappin et al., 2013). Others point to gradually declining, yet elevated levels of depression symptoms in mothers (Miles et al., 2007; Poehlmann et al., 2009) or in both mothers and fathers until at least the infant’s corrected age of 6 months (Pace et al., 2016). In addition, some evidence has been found for the connections between prematurity/low birth weight (LBW) and parental depression to last much longer. For instance, according to Barkmann et al. (2018), very low birth weight predicts elevated levels of parental depressiveness even up to 5 years postpartum. Finally, a recent study by Genova et al. (2022) points to general decrease in depressiveness between 3 and 12 months postpartum in mothers and fathers, although with some differences, both in the severity of depressiveness and in the reduction in depressive symptoms over time, between parents of extremely LBW and very LBW infants.

The increased risk for postpartum depression should raise our particular concern as the links between perinatal parental depression and a child’s mental health and developmental problems later in life have been well documented in preterm children (Cheng et al., 2016; Trumello et al., 2018; Neri et al., 2020; Pisoni et al., 2020), adding to the already complex array of challenges to child development related to biological immaturity, neonatal complications, quality of the early experience, etc. (Aarnoudse-Moens et al., 2009). One of the important factors that has recently received considerable attention from researchers as potentially related to compromised parental mental health (especially in terms of risk of depression), is infant temperament as perceived by the parents.

More and more studies point to preterm birth or its neurological complications as related to less optimal infant temperamental profile (Takegata et al., 2021), or even to “difficult” temperament (Washington et al., 1986; Larroque et al., 2005). Preterm birth is a multifold risk for child development (Wolke et al., 2019). Biological immaturity, medical complications, pain exposure, and exposure to overwhelming sensory input in the NICU are among the risk mechanisms that can alter a child’s neurobehavioral functioning (Als et al., 2004; Feldman, 2009; Valeri et al., 2015; Grunau, 2020). For example, exposure to procedural pain and pain-related stress in neonatal period was found associated with the alterations in brain architecture and function (see Gaspardo et al., 2018, for review), which, in turn, may be related to poorer regulatory competencies in later developmental periods. Montirosso et al. (2016) described epigenetic mechanisms through which early NICU-related stress might be associated with temperamental difficulties at 3 months of age.

Studies using Mary Rothbart (2004, 2011) psychobiological, developmental, and dimensional approach to temperament, defined as “biologically rooted individual differences in reactivity and self-regulation in emotional, activational, and attentional processes” (Fu and Pérez-Edgar, 2015, p. 193), clearly point to specificities of preterm children’s temperament. Noteworthy in this approach is that reactivity is captured by the dimensions of Negative affectivity and Surgency, whereas self-regulation is reflected in the dimension of Effortful control (Orienting/regulation in infancy) (Fu and Pérez-Edgar, 2015). Furthermore, each of the three higher-order dimensions consists of a number of lower-order temperamental traits (dimensions).

In the study by Cosentino-Rocha et al. (2014), preterm birth was associated with higher scores on high-intensity pleasure and perceptual sensitivity and lower scores on discomfort, cuddliness, and attentional focusing in children aged 18 months to 5 years. The study by Tamm et al. (2020) in the group of very preterm infants showed that early MRI-diagnosed brain abnormalities were predictive of lower parental ratings of child’s temperamental features as measured by the Infant Behavior Questionnaire-Revised-Short form (Putnam et al., 2014): High Intensity Pleasure and Vocal Reactivity, High Intensity Pleasure and Cuddliness as well as Fear and Sadness at 3 months corrected age. According to a meta-analysis conducted by Cassiano et al. (2020), preterm children get lower scores in Attentional Focusing and higher scores in Activity dimensions compared to full-term children. Compromised temperamental profile in turn was found to be predictive of long-term behavioral problems (Cassiano et al., 2016, 2019; Lee and Lee, 2017; Martins et al., 2021).

Associations between maternal depressive symptoms and infant behavior and temperament have been broadly documented (e.g., McGrath et al., 2008). However, the study results do not explain the mechanisms which underlie this association, and the direction of the relationship remains unclear (e.g., Murray et al., 1996; Pauli-Pott et al., 2004; Britton, 2011; Eastwood et al., 2012; Aktar et al., 2017). And this is particularly true for preterm infants. Some studies show that the temperamental profile of preterm infants is predictive of maternal depressive symptoms. For example, according to results from Quist et al. (2019), gestational age was predictive of maternal depressive symptoms, but only in interaction with fussiness. Contrary directions were found in studies which point, that maternal depression can alter the perception of child behavior. The study by Voegtline et al. (2010), showed the stability of increased symptoms of depression and anxiety at 2 and 6 months in mothers of late preterm infants, which was related to higher maternal ratings of infant negativity at 6 months.

More research is required to better understand the relationship between parental depression and child temperament. This is especially true in parents of preterm infants due to higher prevalence of depressive symptoms in mothers and fathers, altered temperamental profiles in infants, and numerous challenges in parent-infant interactions related to both parental (e.g., Misund et al., 2016) and infants’ (e.g., Harel et al., 2011; Poehlmann et al., 2011) contributions in this population. Additionally, investigating the association between the course of parents’ depressive symptoms and infant temperament might be of crucial clinical importance, as depression in the postpartum period may have long-term detrimental consequences for parental well-being (Hermens et al., 2004; Vigod et al., 2010; Helle et al., 2015) and child development (Latva et al., 2008; Cheng et al., 2016; Slomian et al., 2019).

*In the current study*, we aimed to analyze the relationship between the intensity of depressive symptoms (depressiveness) in parents of preterm infants and parents’ perceived infant temperament at 3 months corrected age (CA). Furthermore, our intention was to verify whether both the intensity of depressiveness and the infant’s temperamental dimensions, as assessed by parents at 3 months, were predictors of parental scores on the dimension of depressiveness at 6 months of the infant’s CA. Taking into consideration the available research data briefly summarized in the introductory section, and Rothbart’s model of temperament, we hypothesized that:

(1) The level of depressiveness will be higher in mothers than in fathers, both at 3 and 6 months infants’ CA.
(2) The level of depressiveness of both mothers and fathers will decrease between 3 and 6 months of the infant’s CA.
(3) Measures of infants’ temperament as assessed by mothers and fathers will be positively correlated.
(4) Infant’s Negative affectivity at 3 months CA will be positively related to the maternal and paternal level of depressiveness at 3 and 6 months CA, whereas indices of temperamental self-regulation (Orienting/regulation in the case of infants) and Surgency/extraversion at 3 months CA will be negatively related to both maternal and paternal depressiveness at 3 and 6 months CA.

In addition, an exploratory analysis was planned to explain possible contribution of parental depressiveness scores and parent-reported infant’s temperament at 3 months to maternal and paternal levels of depressiveness at 6 months CA.

## Materials and Methods

### General Information

This study was part of a larger longitudinal project on relational and biological antecedents of self-regulatory capacities of preterm infants in the first year of their lives, in which data were collected at 1, 3, 6, and 12 months infants’ CA. For the purposes of the current study, only the data collected at 3 and 6 months will be used as these are specifically targeted at assessing parental levels of depressiveness and infant temperament. Our focus was on families with infants born before the 34th gestational week, hospitalized for at least 7 days in the neonatal unit, as their experiences might considerably differ from those of late preterm infants.

### Participants

A convenience sample of sixty-four infants (30 girls) born before the 34th gestational week in two tertiary care hospitals in Warsaw between July 2008 and February 2010 was enrolled. The parents were invited via written information about the study distributed by the neonatal unit staff just before each infant’s discharge from the hospital. The inclusion criteria also comprised both parents’ consent to participate in all assessment meetings, and parents being above 19 years of age. Infants from multiple pregnancies as well as those born with additional metabolic or genetic syndromes, congenital malformations, or tumors were excluded. The study was approved by the research ethics committee of the Faculty of Psychology, University of Warsaw, and conformed to the Declaration of Helsinki. In the original project, a control group of 31 full-term infants was also included but will not be presented here as our focus is not on comparison between the groups but specifically on the interplay of factors that might explain the intensity and persistence of depressive symptoms in parents of preterm infants, and the possible links with infants’ temperament.

The preterm group consisted of two subgroups in line with the WHO degrees of prematurity: 33 infants met the criteria for extreme prematurity (EPT), and 31 infants were born very or moderately preterm (VPT and MPT, respectively). Four families resigned from the study (3 from the EPT and 1 from the VPT group) by the time the infant reached 3 months CA. Apart from that, one family could not participate in the assessment for a period of 3 months for medical reasons. As no statistically significant correlations were found between infants’ gestational week and either parental depressiveness scores (at 3 and 6 months) or infants’ temperamental dimensions, both groups of preterm infants were merged for further analysis in this study. Group characteristics are presented in Table 1. Mean gestational age of the infants at birth was 28.746 weeks (*SD* = 3.15; *Mdn* = 29), mean birth weight was 1,290.8 g (*SD* = 519.475; *Mdn* = 1,200), mean length of hospitalization was 60 days (*SD* = 34.115; *Mdn* = 34), and the mean number of skin breaking/painful procedures was 91 per hospital stay (*SD* = 95; *Mdn* = 45). The socio-economic status of the families was controlled for, and all the families reported that their financial situation was either average or above average. Most parents had at least 12 completed years of education, and 70% of mothers and 56.7% of fathers had a higher education diploma. Mothers were between 20 and 41 years of age (*M* = 31.5, *SD* = 4.2, *Mdn* = 31), while fathers were between 20 and 52 (*M* = 33.8, SD = 5.26, *Mdn* = 33).

**TABLE 1:** Group characteristics.

	N	%	Mean	SD	Median	Min	Max
Total number of infants	59	100.00					
Girls	30	50.85					
Boys	29	49.15					
Infant’s gestational week at birth			28.75	3.15	29.00	22.00	34.00
Infant’s birthweight in grams			1, 290.80	519.48	1, 200.00	495.00	2, 440.00
Infant’s duration of hospital stay after birth			59.70	34.12	54.00	7.00	147.00
**Prevalence of neurological complications:**							
IVH of at least 3rd grade	8	13.56					
PVL	5	8.47					
Prevalence of retinopathy of prematurity of at least 3rd grade	14	23.73					
Prevalence of necrotizing enterocolitis (NEC)	6	10.17					
Prevalence of chronic lung disease	29	49.15					
Days on mechanical ventilation			10.76	15.84	4.00	0.00	63.00
Number of skin-breaking/stressful procedures during the whole hospital stay			91.45	94.97	45.00	5.00	384.00
Mother’s age (years)			31.37	4.17	31.00	20.00	41.00
Father’s age (years)			33.71	5.33	33.00	20.00	52.00
Mother’s education (number of completed years)			15.73	2.28	17.00	8.00	19.00
Father’s education (number of completed years)			15.10	2.78	17.00	11.00	20.00

*IVH, intraventricular hemorrhage; PLV, periventricular leukomalacia.*

It is worth noting that, at the time when the infants were hospitalized, the two neonatal units adopted various elements of neurodevelopmental care such as special nests surrounding and supporting the infant’s body, blankets shielding an isolette to minimize excessive and abrupt light exposure, etc. The units also offered psychological support for the parents and employed a team of physiotherapists and speech therapists to provide developmentally appropriate care for the infants.

Furthermore, participation in the project as such might have served as an additional supportive measure because it involved two home visits by a nurse and a psychologist (at the infant’s CA of 1 and 3 months), with plenty of time for parents to share their concerns with the study team, and the provision of feedback on the infants’ developmental progress and parent-infant interactions.

### Procedure

As already mentioned, families were first approached at around the time of their infant’s discharge from the hospital. Written informed consent was obtained from all adult participants, and the parents were asked to complete a socio-demographic questionnaire. In addition, the medical records of each child were analyzed by a project leader, a neonatologist, and a neonatal nurse in order to retrieve data on infants’ gestational age at birth, birthweight, days of mechanical ventilation and hospitalization, number of neonatal skin-breaking procedures, and complications of prematurity (see section “Measures”).

At 3 months CA, a home visit was scheduled for each family, at a time convenient for them. The appointments with the families were arranged by a research team member via a phone call, based on prior written consent from both parents. Mothers and fathers were asked to independently rate their infant’s temperament and complete a screening tool for postnatal depression. Information on any changes in the infant’s health status and family socio-economic status was updated. This phase of the project was completed no later than 4 months CA, mostly at 3 months and 15 days.

At 6 months CA families were invited to a baby lab at the Faculty of Psychology of the University of Warsaw and were asked to repeat completion of a postnatal depression screening tool. Apart from that, interviews with parents were also conducted with a focus on each infant’s functioning across a range of typical domains (sleep patterns, feeding, arousal regulation, developmental milestones, and health, etc.). The second visit was supposed to be arranged no later than within 15 days from the time when infants were 6 months CA, and, in fact, the mean CA was 5 months and 29 days (SD = 12.00).

### Measures

#### Socio-Demographic Questionnaire

Data on parental age, level of education (number of completed years of formal education), housing, financial situation, employment, and number of other children in the family were collected.

#### Data From Medical Records

The following data were extracted from the infants’ medical records: birthweight, gestational age, small for gestational age (yes/no), number of days in hospital, number of days on mechanical ventilation, neurological complications (intraventricular hemorrhage/which grade, periventricular leukomalacia, and other), number of skin-breaking procedures during hospital stay, necrotizing enterocolitis (yes/no), retinopathy of prematurity (which grade), bronchopulmonary dysplasia (yes/no), and infection (yes/no), etc.

#### Infant Behavior Questionnaire–Revised

The Polish version of IBQ-R (Gartstein and Rothbart, 2003; Polish adaptation Dragan et al., 2011) is a 186-item parent – report measure of infant temperament based on Rothbart’s approach. It can be used between the ages of 3 and 12 months, and measures 14 temperamental dimensions that load three major factors: Surgency/extraversion (comprising the scales of Approach, Vocal Reactivity, High Intensity Pleasure, Smiling and Laughter, Activity Level, and Perceptual Sensitivity), Negative affectivity (comprising the scales of Sadness, Distress to Limitations, Fear, and Falling Reactivity/Rate of Recovery from Distress), and Orienting/regulation (comprising the scales of Low Intensity Pleasure, Cuddliness, Duration of Orienting, and Soothability). Each item is rated on a 7-point scale (from 1 – never to 7 – always), and parents are asked to report on behaviors observed during the last week. In addition, parents can choose “does not apply” option, and no numerical score is assigned to a given item in such a case. Scale scores are computed as the mean score of all scale items applicable to the child, as reported by the caregiver. Similarly, the score for each of the three major factors is represented by the mean score of the relevant scales. The internal consistency for the 14 temperamental dimensions was performed on a bigger sample of infants and turned out to be satisfactory, with Cronbach’s alphas ranging from 0.73 to 0.89 for maternal ratings, and from 0.71 to 0.90 for paternal ones (Dragan et al., 2011).

#### Edinburgh Postnatal Depression Scale

The Edinburgh Postnatal Depression Scale (EPDS; Cox and Holden, 2003; Polish translation by Bielawska-Batorowicz) is a 10-item self-report measure for identifying the risk of postnatal depression in women, with each item rated on a 4-point scale from 0 to 3 and referring to the last 7 days. The higher the score, the higher the level of depressiveness. Although Brouwers et al. (2001) have confirmed that this measure contains a subscale of depression and a subscale of anxiety, they still recommend the use of a total score, as this seems to be a better measure of both anxiety symptoms and depressive symptoms than when subscales are used. The EPDS has also been widely used in research on fathers but with rather mixed results. Cut points of 9/10 and 12/13 have been suggested to identify a risk of minor vs. major depression. More recently, a cut-off value of 11 or higher has been found to maximize combined sensitivity and specificity (Levis et al., 2020). In the current study, we will use this measure as a continuous variable representing the level of a subject’s depressiveness.

The Polish version of EPDS had high internal consistency for the assessments of mothers at 3 months CA, Cronbach’s alpha = 0.88, and acceptable at 6 months CA, Cronbach’s alpha = 0.78. The internal consistency for the assessment of fathers was also high at 3 months CA, Cronbach’s alpha = 0.80, and lower than acceptable at 6 months, Cronbach’s alpha = 0.67.

### Statistical Analyses

First, we used a non-parametric test to compare mothers’ and fathers’ EPDS scores at 3 months CA. Second, we tested whether mothers’ EPDS scores changed from 3 to 6 months CA. Third, we compared mothers’ and fathers’ ratings of infants’ temperament at 3 months CA. Then, we performed correlation analysis to search for possible predictors of mothers’ EPDS scores. In order to determine whether infants’ perceived temperament at 3 months CA predicted mothers’ EPDS scores at 6 months CA, controlling for mothers’ EPDS scores at 3 months CA, we conducted a regression analysis. Due to the low internal consistency of EPDS at 6 months CA for fathers, analyses with those scores were not performed.

## Results

### Mothers’ and Fathers’ Scores on the Edinburgh Postnatal Depression Scale and Infant Behavior Questionnaire–Revised

Table 2 presents descriptive statistics for EPDS and IBQ-R scores. Mothers scored significantly higher than fathers on the EPDS at 3 months CA, *U* = 1344.00, *z* = −2.01, *p* = 0.044. A related-samples Wilcoxon signed rank test revealed that there was a statistically significant difference between mothers’ EPDS scores at 3 and 6 months postpartum, *T* = 417.00, *z* = −2.49, *p* = 0.013. Mothers’ EPDS scores decreased between 3 and 6 months of children’s CA.

**TABLE 2:** Mothers’ and father’s EPDS scores at 3 and 6 months CA and IBQ-R scores at 3 months CA: Descriptive statistics.

	Mdn	Min	Max	Q1	Q3
EPDS M 3 MCA	6.00	0.00	27.00	4.00	10.00
EPDS F 3 MCA	5.00	0.00	22.00	3.00	9.00
EPDS M 6 MCA	5.00	0.00	19.00	4.00	8.00
EPDS F 6 MCA	5.00	0.00	14.00	3.00	6.00
IBQ-R Surgency/extraversion M	3.90	1.99	5.54	3.47	4.39
IBQ-R Negative affectivity M	3.49	2.79	4.70	3.22	4.75
IBQ-R Orienting/regulation M	4.81	3.38	6.18	4.34	5.15
IBQ-R Surgency/extraversion F	3.84	2.48	5.49	3.36	4.28
IBQ-R Negative affectivity F	3.39	2.45	4.22	3.17	3.68
IBQ-R Orienting/regulation F	4.52	3.08	5.88	4.26	4.96

*EPDS, Edinburgh Postnatal Depression Scale; MCA, months corrected age; M, mother, F, father; IBQ-R, Infant Behavior Questionnaire-Revised.*

There were no significant differences between mothers’ and fathers’ assessments of their infants’ Negative affectivity or Surgency/extraversion; both *p*s > 0.05. Regarding Orienting/regulation, there was a trend approaching significance, suggesting higher scores of mothers than fathers, *U* = 1314.00, *z* = −1.89, *p* = 0.059.

### Correlations Between Mothers’ and Fathers’ Edinburgh Postnatal Depression Scale and Infant Behavior Questionnaire–Revised Scores

Table 3 presents the results of correlation analyses for mothers’ and fathers’ depressive symptoms (EPDS scores) and their perceptions of their infants’ temperament (IBQ-R scores). The correlations between mothers’ and fathers’ ratings of their infants’ temperament were either non-significant or very weak. Mothers’ ratings of their infants Negative affectivity and Orienting/regulations were correlated with their EPDS scores. There was no such association for fathers.

**TABLE 3:** Correlations (τ_b_) between mothers’ and fathers’ EPDS scores at 3 and 6 months corrected age and IBQ-R scores at 3 months corrected age.

	EPDS M 3 MCA	EPDS F 3 MCA	EPDS M 6 MCA	IBQ-R Surg/extr M	IBQ-R Neg aff M	IBQ-R Orient/reg M	IBQ-R Surg/extr F	IBQ-R Neg aff F
EPDS F 3 MCA	0.07							
EPDS M 6 MCA	0.43**	0.10						
IBQ-R Surg/extr M	0.00	−0.03	−0.07					
IBQ-R Neg aff M	0.09	0.06	0.19*	0.09				
IBQ-R Orient/reg M	−0.13	0.02	−0.23*	0.35**	−0.05			
IBQ-R Surg/extr F	0.01	−0.04	0.02	0.24*	−0.07	0.05		
IBQ-R Neg aff F	−0.20*	0.04	−0.13	−0.11	0.12	0.05	0.10	
IBQ-R Orient/reg F	0.02	−0.07	−0.11	0.24**	−0.01	0.15	0.40**	0.00

EPDS, Edinburgh Postnatal Depression Scale; MCA, months corrected age; M, mother; F, father; IBQ-R, Infant Behavior Questionnaire-Revised; Surg/extr, surgency/extraversion; Neg aff, negative affectivity; Orient/reg, orienting/regulation. Bold text indicates a statistically significant correlation; *p < 0.05; **p < 0.001.

Notably, no statistically significant correlations were found between mothers’ and fathers’ EPDS scores at 3 months CA.

As the IBQ-R Negative affectivity and Orienting/regulation scores as assessed by mothers, and the mothers’ EPDS scores at 6 months CA, were significantly correlated, we conducted regression analyses with these two dimensions of infant temperament perceived by the mothers at 3 months CA as predictors of the mothers’ depressive symptoms at 6 months CA.

### Regression: Infant Temperament Predicting Depression Symptoms

We tested whether infants’ Negative affectivity and Orienting/regulation at 3 months CA as perceived by the mother predicted mothers’ depressive symptoms at 6 months CA, controlling for mothers’ depressive symptoms at 3 months CA (see Table 4).

**TABLE 4:** Regression: maternal EPDS scores, infant negative affectivity, and infant Orienting/regulation at 3 months as predictors of EPDS scores at 6 months.

	Coefficients							
Model	B	B SE	Beta	*p*	95% CI for *B*		Collinearity statistics	
					LL	UL	Tolerance	VIF
(Constant)	2.76	0.68		0.000	1.39	4.12		
ESDP 3 m.	0.49	0.07	0.66	0.000	0.34	0.63	1.00	1.00
(Constant)	4.55	4.63		0.330	–4.73	18.83		
ESDP 3 m.	0.44	0.07	0.60	0.000	0.30	0.58	0.95	1.05
Neg aff 3 m.	1.77	0.93	0.18	0.063	–0.10	3.64	0.97	1.03
Orient/reg	–1.59	0.65	–0.23	0.018	–2.89	–0.28	0.97	1.03

*EPDS, Edinburgh Postnatal Depression Scale; Neg aff, negative affectivity; Orient/reg, orienting/regulation; CI, confidence interval; LL = lower limit; UL = upper limit; VIF, variance inflation factor.*

In the first step, we entered the control variable: mothers’ scores on the EPDS when the infant was 3 months CA. These scores were significantly positively associated with EPDS scores at 6 months CA, and explained 44% of variance, *R*^2^ = 0.438.

In the second step, we entered the IBQ-R Negative affectivity scores and the IBQ-R Orienting/regulation at 3 months CA. The relation between Negative affectivity at 3 months CA and EPDS scores at 6 months CA was not statistically significant, *p* > 0.05. The IBQ-R Orienting/regulation scores at 3 months CA were significantly positively associated with the EPDS scores at 6 months. Orienting/regulation scores at 3 months CA predicted lower EPDS scores at 6 months CA. The overall model was statistically significant and explained 53% of variance in EPDS scores at 6 months CA, *R*^2^= 0.53, *F*(3, 54) = 19.87, *p* < 0.001.

## Discussion

This study aimed to examine the associations between the levels of parental depressive symptoms and preterm infants’ parents – reported temperament at 3 months, and the risk of maternal and paternal depression at 6 months postpartum. We found an association between self-reported depressive symptoms at 3 months and infant temperament assessment in mothers. Moreover, maternal depressiveness combined with the infant’s temperamental characteristics assessed by mothers at 3 months turned out to be predictive of depressiveness scores at 6 months.

In line with the large body of research, we found higher levels of depressiveness in mothers compared to fathers at 3 months postpartum. It should be emphasized, however, that we have measured depressiveness which is not equivalent to identifying clinically significant depression. The EPDS is a screening tool, not a diagnostic one. Our participants represented a full range of scores with the mean far below the suggested cut point for the risk of major depression. Further studies are, therefore, needed on groups of preterm infants’ parents with clinical diagnosis of mood disorders.

Moreover, contrary to the results of other authors (Neri et al., 2020; Thiel et al., 2020), we have not found a statistically significant correlation between maternal and paternal depressiveness scores. In addition, due to the low internal consistency of EPDS for the assessment of fathers at 6 months, these results could not be used in the analyses. This, in turn, prevented us from checking correlations with maternal depressiveness scores at 6 months, and made a comparison of paternal levels of depressiveness at 3 and 6 months ineligible. Despite our problems with internal consistency of EPDS for fathers at 6 months CA, one of the reasons for this apparently surprising result of not finding correlations between maternal and paternal scores at 3 months CA might be the nature of EPDS as such. Further studies should address this issue with the item by item analysis of maternal and paternal scores on bigger samples. Although EPDS was validated as a measure of postpartum mood in men (Matthey et al., 2001), our results add to the growing literature which emphasizes the need for using gender-specific tools in screening for perinatal depression (Carlberg et al., 2018; Walsh et al., 2020; Yogman, 2021). More and more authors point to the specificity of paternal depression and paternal depressive symptoms, which are not included in EPDS, e.g., irritability, abnormal illness behaviors, heightened anxiety, or addictions (Kim and Swain, 2007; Baldoni and Giannotti, 2020; Walsh et al., 2020; Garfield et al., 2021). Using gender-specific tools could shed more light on the specificity of depressive symptoms in mothers and fathers of preterm infants.

The level of postpartum depressiveness in mothers in our sample was higher at 3 than at 6 months. This is in line with studies showing that the severity of maternal depressive symptoms decreases over time (Miles et al., 2007; Pace et al., 2016; Garfield et al., 2021). However, it is noteworthy that despite the fact that the risk of depression tends to decline over time in the majority of women, about 30% of mothers affected by postpartum depression remain depressive throughout the first year of the child’s life and beyond (Vliegen et al., 2014). The percentage of mothers who meet the criteria of depression many months after delivery turned out to be even higher in women with depression in life history. The risk for depression has been proven to be higher in mothers of preterm infants compared to mothers of full-term infants not only shortly after delivery but also throughout the following months (Vigod et al., 2010; Neri et al., 2020). Moreover, although the directions of these associations are complex, researchers point to the links between preterm infants mothers’ postpartum depression and the chronicity of maternal mental health problems (Miles et al., 2007; Pace et al., 2016), paternal depression (Carlberg et al., 2018; Neri et al., 2020), and less than optimal child developmental outcomes (Cheng et al., 2016; Narayanan and Nærde, 2016). For this reason, investigating factors predictive of the persistence of maternal depressiveness is of crucial importance for clinical practice and early intervention addressed to families of preterm infants. Further studies are needed to disentangle possible links between trajectories of maternal and paternal depressiveness and all the different factors related to the severity of infant’s prematurity and the comorbid medical complications.

We did not find any significant differences in infants’ temperamental characteristics obtained from mothers and fathers. This result is in line with studies showing similarities between maternal and paternal assessments of infant’s temperament (Sechi et al., 2020; Vismara et al., 2021). The reliability of parental perceptions may indicate that regardless of the specificity of and possible differences in experiences mothers and fathers shared with their infants, the parental assessments of the infant’s temperament were related to the child’s actual behaviors. On the other hand, the correlations between maternal and paternal assessments of infants’ temperament were rather weak in our study, quite contrary to previous research findings (Dragan et al., 2011). This may point to a presumably complex nature of parental perceptions of preterm infants’ temperament, and calls for including observational or at least independent measures of temperament when assessing this at-risk group.

According to our results, Orienting/regulation, but not Negative affectivity, as assessed at 3 months predicted maternal depressiveness at 6 months, which means that our hypothesis as to the links between maternal perceptions of infant temperament and self-reported depressiveness has only partly been supported. In accordance with our hypothesis, heightened scores in EPDS, combined with assessing the child as low on Orienting/regulation at 3 months, turned out to be predictive of mothers’ depressiveness at 6 months. At the same time, we haven’t found any support for Negative affectivity, as assessed at 3 months, to be a predictor of maternal depressiveness at 6 months. This is quite in contrast to the results obtained by Sechi et al. (2020) for parents of full-term infants, where maternal depression and anxiety symptoms were positively correlated with Negative affectivity, but not with Orienting/regulation, at 3 and 12 months.

Our results point to behaviors that may be particularly significant for mothers of preterm infants in the context of postpartum depression. Data on early preterm infants’ temperament indicate that in the first weeks and months of life, preterm infants may be less rhythmic, more difficult to soothe, more withdrawn, and spend less time in alert states, which may hinder rewarding experiences in early parent-infant interaction (Eckerman et al., 1995; Hughes et al., 2002). The Feldman and Eidelman (2007) confirmed a slower maturation of preterm infants’ autonomic nervous system and decreased capability to maintain alert states. The authors described the double interactional risk linked to the attention regulation in preterm infants as lower infants’ interactional availability and compromised maternal ability to coordinate their interactional behaviors with the fragile infant. Maternal depressiveness turned out to be associated with both mothers’ interactional behaviors and infants’ vagal tone. These results may show that preterm birth, maternal depressiveness, and mothers’ as well as infants’ interactional behaviors constitute a multitude of factors that put preterm children and their parents at risk.

Our results add to this knowledge and confirm the complexity of the mechanisms underlying early mood problems in parents of preterm infants. They contribute to the literature on risk mechanisms of postpartum depression in parents of preterm infants by linking parental mood with the temperamental profile of preterm infants. They also show possible links between preterm infant temperament and the chronicity of postpartum depressiveness. Our results further suggest that special clinical attention should be given during screening to those mothers of preterm infants whose depressiveness at 3 months co-occurs with perceiving the infant as difficult to soothe, showing little enjoyment at being held in the arms of the adult, and having problems with maintaining alert states or attending to/interacting with people and objects for extended time.

There are studies that indicate early interventions focused on parental perception of the preterm infant’s behaviors, as well as on sensitive caregiving, are effective in supporting parental assessment of a child’s emotionality and soothability (Landsem et al., 2020). Offering early support to mothers who perceive their infants as temperamentally challenging, and whose rates of depressiveness are elevated, might prevent them from further mood problems.

We did not find a significant relationship between paternal depressiveness and child temperament assessment at 3 months CA. This is in contrast with studies in which paternal depression was related to child temperamental characteristics (Hanington et al., 2010). In research using Rothbart’s approach to temperament, depression symptoms in fathers were found to be significantly related to the assessment of distress (Ramchandani et al., 2011) and negative affectivity (Sechi et al., 2020) in 3-month-old infants. Those studies, however, were conducted on the group of full-term infants’ fathers. Far less is known about the links between depressive symptoms and infant temperament assessment in fathers of preterm infants. Due to the low internal consistency of EPDS for fathers, we could not verify our hypotheses referring to the links between paternal depressiveness at 3 and 6 months, and child temperamental assessment. This remains a direction for future studies. However, as we found similarities between maternal and paternal perceptions of a child’s temperament and the temperamental assessment correlated with the severity of depressive symptoms in mothers, we hypothesize that interaction between parent-specific caregiving and child’s temperamental characteristics may be related to the risk for postpartum depression. This hypothesis requires further investigation.

Future studies should also address a contribution of child gender to parental perceptions of preterm infants’ temperament, which we haven’t analyzed as this is outside of the scope of the present study. Research with full-term children points to an infant’s gender as a significant predictor of parental assessment of their temperamental characteristics (Else-Quest et al., 2006; Sechi et al., 2020). Far less is known in this respect about preterm infants. In the study by Pesonen et al. (2006), infant perinatal status turned out to be significant for parental assessments of a child’s temperament regardless of the birth term. The child’s gender did not differentiate parental perceptions either. One might hypothesize that perinatal status, which has a strong impact on early infant and parent experiences, may have more impact on the maternal and paternal assessment of infant behavior and temperament than a child’s gender *per se*. This hypothesis may be a direction for future studies.

Another promising direction for further research is the analysis of possible links between the severity and complications of prematurity, and maternal/paternal perceptions of infant temperament and parental mental health. This is especially relevant in light of recent findings on early programming of preterm infants’ temperament, via gene methylation processes, due to neonatal exposure to pain and stress related to medical procedures and treatment in the Neonatal Intensive Care Unit (NICU) (Cassiano et al., 2016; Montirosso et al., 2016). Furthermore, interconnections between the severity of preterm infant medical conditions and parental mental health have already been established (Agostini et al., 2014; Carson et al., 2015; Neri et al., 2020). Neonatal data that we have collected and included in our sample characteristics clearly point to the numerous challenges that the infants under study were exposed to, not to mention an additional emotional burden for the parents. A question arises as to the possible role of infants’ medical conditions in explaining the links between infant temperament and mothers’ and fathers’ depressiveness. Future studies should make an attempt at disentangling this important issue.

## Limitations

Finally, several limitations of our study should be addressed. First of all, the size of our sample was rather small, with numerous implications for data analysis and the results. For example, with smaller samples the assumption of variables’ distribution normality is often violated, preventing the use of more powerful statistical tests. In addition, a small sample size hinders the inclusion of more variables in the regression analysis, thus limiting the possibilities for testing different, more complex models of relationships among the variables. Furthermore, our sample was characterized by relatively high rates of parents with higher education and medium to high socio-economic status. Hence, the results cannot be generalized to samples with lower SES and education. Apart from that, the study design required the active participation of both parents, which is not a limitation in itself but does narrow the possibilities of extending our results to single-parent families or families with less involved fathers. Although this was outside of our study’s scope, a lack of the inclusion of infants’ medical conditions in the analyses can certainly be treated as a limitation in generalizing the results. Another limitation is the choice of EPDS as a measure of depressiveness in the case of fathers. As already mentioned, this screening tool, although widely used in other studies with fathers as participants, may not be well suited for discerning specific features of depressive symptoms that are characteristic for men, such as acting out, aggression, psychosomatic complaints, etc. This should be a focus of attention in future studies, with a possible choice of screenings specifically addressed to fathers (Baldoni and Giannotti, 2020), or the additional inclusion of other measures of depressive symptoms such as Center for Epidemiologic Studies Depression Scale (CES-D) or Patient Health Questionnaire-9 (PHQ-9). Furthermore, no information on the levels of depressiveness before childbirth was included in our study, not to mention the history of parental mental health in general. Last but not least, detailed information on the participants’ current use of psychotherapy, parental support groups, and other sources of emotional support should have been included.

## Conclusion and Implications

Our study points to the importance of taking into account maternal mood along with perceptions of preterm infants’ temperament as early as at the age of 3 months CA in the analysis of mothers’ level of depressiveness later in the first year of the child’s life. Complex, transactional relationships between an infant’s temperament as assessed by the parents and parental mental health in the face of prematurity can be postulated and require further investigation. With bigger, multi-site cohorts of preterm infants and their parents and the newest statistical methods, longer-term trajectories of the interplay between the intensity of depressive symptoms and parental perceptions of child reactivity and self-regulation can and should be studied. Further studies should also assume a more fine-grained approach to temperament and, with bigger samples, assess more detailed temperamental profiles of preterm infants on all 14 lower-order dimensions, with closer attention to the severity of prematurity.

In terms of methodology, our results raise a concern regarding the use of the EPDS for measuring depressiveness in fathers of preterm infants. Thus, we add to the already existing call for more gender-sensitive screening tools for the risk of depression.

From a practical, clinical point of view, the associations we have found between infant temperament and maternal depressiveness may be of particular importance for designing assessment, prevention, and intervention measures specifically addressed to parents of prematurely born children. Considering the additional risks to child development and family well-being impinged by compromised parental mental health, psychological support for mothers and fathers of preterm infants should be offered far beyond an infant’s stay in the NICU.

## Data Availability Statement

The raw data supporting the conclusion of this article will be made available by the authors, without undue reservation.

## Ethics Statement

The studies involving human participants were reviewed and approved by Research Ethics Committee of the Faculty of Psychology, University of Warsaw. Written informed consent to participate in this study was provided by the participants’ legal guardian/next of kin.

## Author Contributions

GK, EK, and AN: study concept, design, data collection, data analysis, writing the manuscript, critical review, and approved the submitted version.

## Conflict of Interest

The authors declare that the research was conducted in the absence of any commercial or financial relationships that could be construed as a potential conflict of interest.

## Publisher’s Note

All claims expressed in this article are solely those of the authors and do not necessarily represent those of their affiliated organizations, or those of the publisher, the editors and the reviewers. Any product that may be evaluated in this article, or claim that may be made by its manufacturer, is not guaranteed or endorsed by the publisher.
